# Feature-specific reactivations of past information shift current neural encoding thereby mediating serial bias behaviors

**DOI:** 10.1371/journal.pbio.3002056

**Published:** 2023-03-24

**Authors:** Huihui Zhang, Huan Luo

**Affiliations:** 1 School of Psychological and Cognitive Sciences and Beijing Key Laboratory of Behavior and Mental Health, Peking University, Beijing, China; 2 PKU-IDG/McGovern Institute for Brain Research, Peking University, Beijing, China

## Abstract

The regularities of the world render an intricate interplay between past and present. Even across independent trials, current-trial perception can be automatically shifted by preceding trials, namely the “serial bias.” Meanwhile, the neural implementation of the spontaneous shift of present by past that operates on multiple features remains unknown. In two auditory categorization experiments with human electrophysiological recordings, we demonstrate that serial bias arises from the co-occurrence of past-trial neural reactivation and the neural encoding of current-trial features. The meeting of past and present shifts the neural representation of current-trial features and modulates serial bias behavior. Critically, past-trial features (i.e., pitch, category choice, motor response) keep their respective identities in memory and are only reactivated by the corresponding features in the current trial, giving rise to dissociated feature-specific serial biases. The feature-specific automatic reactivation might constitute a fundamental mechanism for adaptive past-to-present generalizations over multiple features.

## Abstract 2

The regularities of the world entail an intricate interplay between past and present. This neural imaging study demonstrates that the past automatically shapes the present through the co-occurrence of past information reactivation and encoding of current information.

## Introduction

The regularities and recurrences of our world render the past always relevant to the present [1]. Indeed, perceptual experience and decision-making at any moment are constantly intertwined with previous information and likewise impact the future [2], a characteristic allowing for perceptual stability and adaptive optimization. The past-to-present influences could occur automatically and span a relatively long time scale. For example, the perceived feature in the current trial (e.g., location, orientation, and category) tends to be systematically shifted by that in the previous trial, even though trials are independent of each other and several seconds apart, namely the “serial bias” effect [3–10].

Crucially, the information contained in the past trial is not simple but rather rich and manifold, encompassing multitudes of features at various levels, e.g., physical properties, abstract categories, and response actions. Different features are found to be associated with different serial biases, in attractive or repulsive directions, even within the same task [7,8,11–13]. A two-stage model employing efficient coding and Bayesian inference reconciles the repulsive and attractive serial bias in perceptual decisions by, respectively, linking them to sensory adaptation and post-perceptual Bayesian inference ([5,14]; but see [15–18]). However, the neural mechanism underlying the multifeature serial bias remains obscure.

For serial bias to occur, previous trial information must leave memory traces that shape perception and decision-making in the current trial, a process that presumably involves memory reactivation and operation. It is known that long-term memory can be reactivated to enter and potentially impact working memory (WM) when necessary [19,20]. Interestingly, recent WM studies suggest that although information could be retained in an “activity-silent” state [21–27], memory operations still rely on the activation of neural representations [28,29]. Accordingly, previous studies have demonstrated that past-trial information is reactivated when processing new inputs, a process that potentially contributes to serial bias [30–32]. Moreover, a recent study observed that previous information remained in activity-silent traces reappeared before the stimulus, and the reactivation strength correlates with the strength of serial bias [33]. This is in line with computational modeling studies that advocate a purely “activity-silent” synaptic plasticity mechanism for serial bias, which can be enhanced by past information reactivation [33–35]. As for the direction of serial bias, i.e., attractive or repulsive, the neural basis remains debated. Using consecutive trials containing the same or orthogonal orientations, one fMRI study shows neural evidence for an attractive bias in early visual cortex [36]. In contrast, recent MEG and fMRI studies [37,38] indicate a repulsive bias during current sensory encoding in early visual cortex, but no evidence for an attractive bias. Based on computational modeling, they propose a higher-level attractive integration [38].

Here, we aim to understand the dynamic neural mechanisms of how multiple features in the previous trial confront and modify the present, from which the feature-specific serial bias arises. In two experiments, participants performed an auditory categorization task with their brain activities recorded using electroencephalography (EEG). First, behavioral results exhibit concurrent component-specific serial biases, i.e., repulsive for tone pitch and motor response and attractive for category choice. Importantly, the neural representations of features in the previous trial—pitch, category choice, and motor response—are reactivated and emerge simultaneously as the corresponding features in the present trial. Most crucially, the current neural representations exhibit behaviorally congruent shifts, i.e., being attracted or repulsed from the past in a feature-specific way, which further correlates to serial bias behavior.

Taken together, the past is intimately mingled with the present via a feature-specific reactivation mechanism, whereby previous information lingering in memory is triggered by the corresponding feature occurrence so that their co-emergence in time leads to serial bias.

## Results

### Task paradigm and feature-specific behavioral serial bias

**Fig 1:**
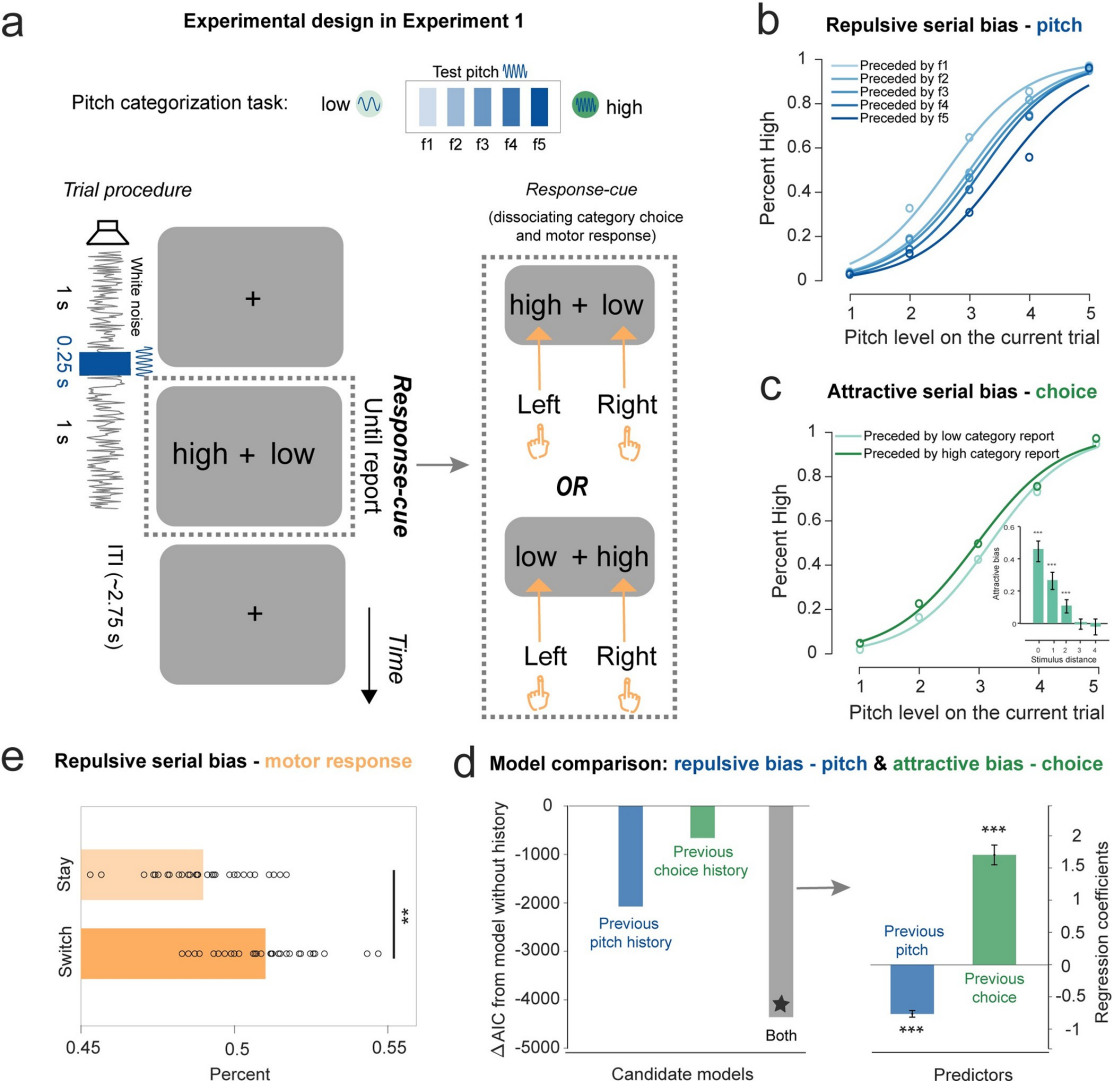
Task paradigm and behavioral serial bias in Experiment 1. (**A**) Auditory categorization task paradigm. Upper: Pitch of the pure tone stimulus was pseudorandomly selected from 5 fixed frequencies (f1, f2, f3, f4, and f5) between 180 Hz (low category; light green) and 360 Hz (high category; dark green). Lower: In each trial, participants categorized a given 0.25-s pure tone (dark blue; selected from f1, f2, f3, f4, and f5) embedded in a 2.5-s sustained white noise stream (grey line) into “high” (closer to 360 Hz) or “low” (closer to 180 Hz) category. After the pure tone, a response–cue screen appeared, based on which participants used the corresponding hand to made responses (right inset). (**B**) Pitch serial bias (aggregate results across participants). “High” category choice percent as a function of the current pitch, for different pitch in previous trial (Light-to-dark color lines denote low-to-high pitches). Each circle represents aggregated data for each condition. Solid lines represent the logistic regression fits. (**C**) Category serial bias (aggregate results across participants). “High” category choice percent as a function of the current pitch, for different category choice in the previous trial (light green: low category choice; dark green: high category choice). The subpanel shows how the category choice serial bias is modulated by the previous-current pitch distance. Error bars represent 95% confidence interval. (**D**) Model comparison results. Left: ΔAIC of Model 2 (blue; current trial + previous pitch), Model 3 (green; current trial + previous category choice), and Model 4 (grey; current trial + previous pitch + previous category choice), compared to Model 1 (current trial only). Right: Regression coefficients for previous pitch (blue) and previous category choice (green) extracted from the winning model (Model 4, * in model comparison). Error bars represent 95% confidence interval. (**E**) Motor response serial bias (left or right hand). “Switch” trials (dark orange): percentage of trials that are different from previous trial in motor response. “Stay” trials (light orange): percentage of trials that are the same as previous trial in motor response. Each circle represents individual participants. (**: *p* < 0.01, ***: *p* < 0.001). Data supporting this figure found here: https://osf.io/4cwv7/?view_only=3a4885189ebf46aaacf05ef109821d03.

In Experiment 1, 30 participants performed an auditory pitch categorization task with their 64-channel EEG activities recorded, by pressing buttons to indicate the category (“high pitch” or “low pitch”) of a pure tone embedded in a sustained white noise (Fig 1A). The pitch of the tone stimulus was pseudorandomly selected from 5 fixed frequencies between 180 Hz and 360 Hz (f1, f2, f3, f4, and f5), which were individualized to normalize task difficulty across participants (see details in Methods). Moreover, to dissociate category choice and motor response, a response–cue frame appeared after the tone stimulus, based on which participants used the corresponding hand to make choices. Note that participants learned the definition of high- and low-pitch categories in a pretest, i.e., listening to 180 Hz and 360 Hz pure tones (S1 Fig).

As shown in Fig 1B and 1C, both pitch and category choice exhibited the serial dependence effect, yet in different directions. Specifically, the pitch of the current tone tends to be perceived away from that of the preceding pitch, i.e., repulsive serial bias (Fig 1B). For example, the same f3 on the current trial was more likely categorized into the “high” category when preceded by lower pitch (light blue) compared to when preceded by higher pitch (dark blue). In contrast, the categorization performance displayed an attractive serial bias, such that the same f3 on the current trial was more likely to be reported as the “high” category when the previous choice was also “high” (Fig 1C). Moreover, this attractive bias decreased as the distance between previous and current pitches increased (M_0_ = 0.46, 95% CI_0_ = [0.38, 0.51]; M_1_ = 0.27; 95% CI_1_ = [0.21, 0.32]; M_2_ = 0.11, 95% CI_2_ = [0.06, 0.14]; M_3_ = −0.00, 95% CI_3_ = [−0.038, 0.026]; M_4_ = −0.02, 95% CI_4_ = [−0.073, 0.026]; ps < 0.001 for distance ≤ 2), suggesting it is not a repetitive bias caused by lapsing.

Four generalized linear mixed-effects models (GLMMs) were built to account for the behavioral performances. Model 1 assumes that the category choice only depends on the current pitch and a constant bias (base model; no serial bias). Models 2 and 3 consider additional contributions from previous pitch or previous category choice, respectively. Model 4 takes account of contributions from both pitch and category choice in the preceding trial. Models 2 to 4 were evaluated against Model 1 by comparing the Akaike information criterion (AIC) values. As shown in Fig 1D (left), all the three models were better than Model 1 (Model 2: ΔAIC = −2,079; Model 3: ΔAIC = −657; Model 4: ΔAIC = −4,369), indicating the influence of past-trial information on current perception. Moreover, Model 4 outperformed Model 2 and Model 3, supporting that pitch and category choice history together affect the current category decision. Importantly, as shown in Fig 1D (right panel), Model 4 showed negative coefficients for past-trial pitch (mean = −0.79; 95% CI = [−0.89, −0.69]; t(58,002) = −15.44, *p* < 0.001, one-sample *t* test), but positive coefficients for past-trial category choice (mean = 1.70; 95% CI = [1.41, 1.99]; t(58,002) = 11.59, *p* < 0.001, one-sample *t* test), confirming the repulsive and attractive serial bias for pitch and category choice, respectively. Moreover, the same analysis after excluding trials preceded by f1 and f5, the two pitches associated with unambiguous categories, showed the same results (S4A, S4B and S4C Fig), supporting that the repulsive and attractive serial biases for pitch and category choice do not just arise from the preceding unambiguous pitches.

Furthermore, the motor response, which is designed to be independent of category choice, also exhibited serial bias, in a repulsive manner (Fig 1E). Specifically, the “switch” motor response probability across consecutive trials was greater than non-switching (“Stay”) (“Switch”: mean = 0.51, SD = 0.016; “Stay”: mean = 0.49, SD = 0.016; “Switch” versus “Stay”: paired-sample *t* test, t(29) = 3.49, *p* = 0.0015).

Together, participants’ perceptual decision tends to be concurrently shifted by multiple features on the preceding trial, being repulsed from the previous pitch while attracted to prior category choice.

### Decoding multiple features of the current trial

We first performed a time-resolved multivariate decoding analysis [27,39–41] to examine the neural representations of multiple features of the current trial—pitch, category, and motor response. A linear regression analysis was used to fit the neural dissimilarity to feature dissimilarity, and the time-resolved regression coefficients denote the decoding performance, for each feature, at each time point and in each participant (see details in Methods).

**Fig 2:**
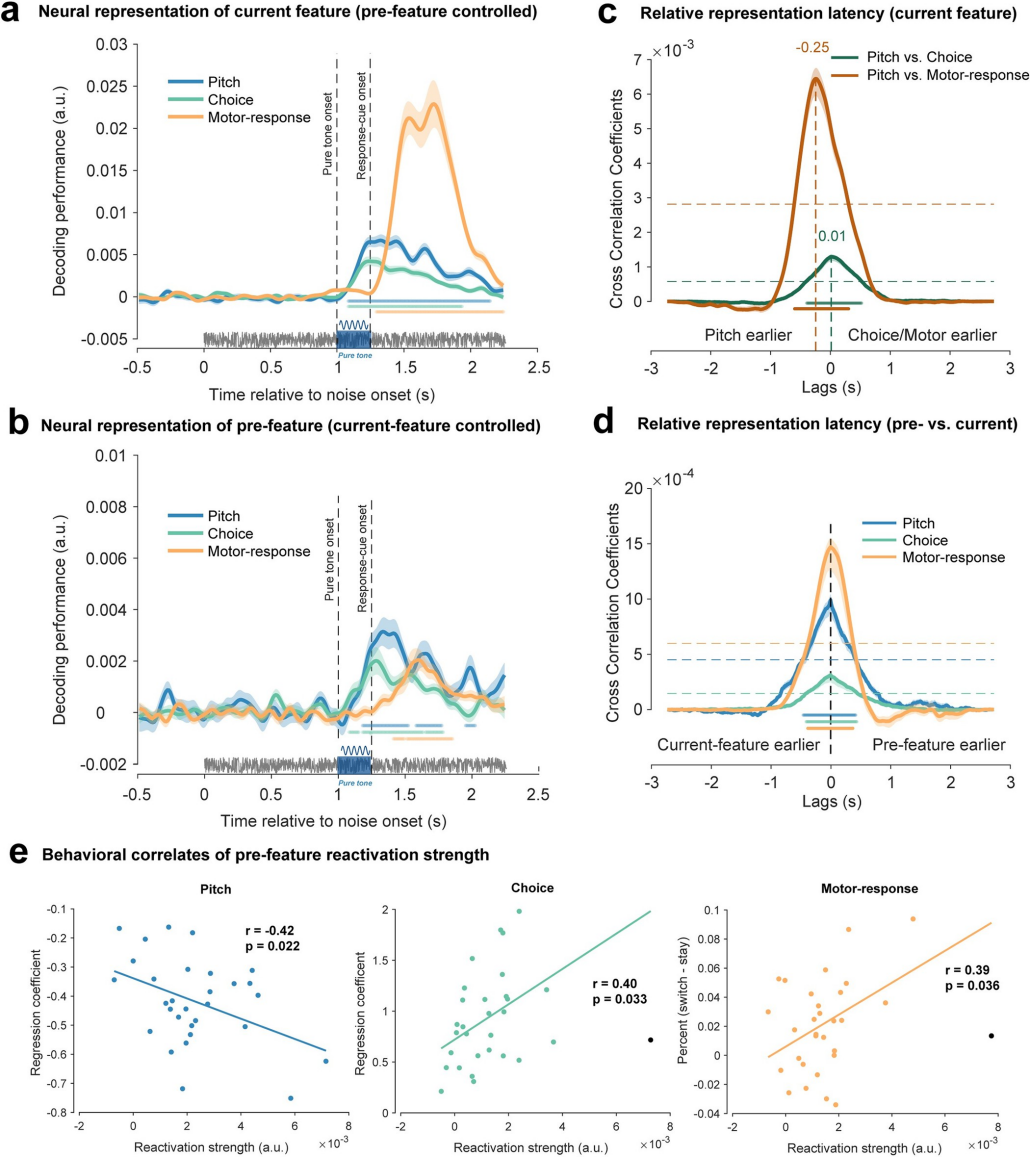
Co-occurrence of past-trial neural reactivations and neural encoding of current-trial features. **(A)** Grand average decoding performance for current-trial features as a function of time following the sustained white noise, for pitch (blue), category choice (green), and motor response (orange). The pure tone (blue rectangle) was embedded in a 2.25-s sustained white noise (grey horizontal line). Vertical dashed lines from left to right denote the tone onset and response cue frame, respectively. Horizontal colored lines denote significant temporal clusters (cluster-based permutation test, *p* < 0.001, one-sided, corrected) for each feature. Shadows represent SEM. (**B**). The same as A but for past-trial features. Note that the past-trial decoding is performed by fixing the current-trial feature. (**C**). Cross-correlation coefficients as a function of temporal lag for each current-trial feature pair (green: pitch vs. category; brown: pitch vs. motor response). Shadow represents 95% confidence interval (bootstrapping, *N =* 5,000). Horizontal colored lines denote significant temporal clusters (cluster-based permutation test, one-sided, corrected, *p* < 0.05) for each feature pair. Horizontal dashed lines represent the 95% permutation threshold (corrected for multiple comparison). Vertical dashed line indicates the significant peak indexing the relative activation lag. (**D**) Cross-correlation coefficients between past-trial reactivation and current-trial neural response, as a function of temporal lag, for each feature (blue: pitch; green: category choice; orange: motor response). The peak at 0 ms temporal lag (vertical dashed line) supports the co-occurrence of past-trial reactivation and current-trial neural response. (**E**) Correlation between past-trial reactivation strength and serial bias behavior across participants (left: pitch; middle: category choice; right: motor response). Reactivation strength is calculated as the mean decoding performance across significant clusters in B. Serial bias behaviors for pitch, category choice, and motor response are indexed by regression coefficients of past pitch, past choice, and difference between “switch” and “stay,” respectively. Each dot represents individual participant, with black dots denoting excluded participants as outliers. Data supporting this figure found here: https://osf.io/4cwv7/?view_only=3a4885189ebf46aaacf05ef109821d03.

As shown in Fig 2A, the pitch (blue) and reported category (green) of the current trial could be decoded shortly after the pure tone (cluster-based permutation test, *p* < 0.001, one-sided, corrected; pitch: 84 to 1,144 ms after tone onset; category: 84 to 934 ms after tone onset). Furthermore, the motor response decoding (Fig 2A, orange) occurred after the response cue frame (cluster-based permutation test, *p* < 0.001, one-sided, corrected; motor response: 44 to 994 ms after response cue frame). This is well expected since participants could only determine the responding hand after the response cue.

Overall, multiple features of the current trial could be successfully decoded from the neural response, i.e., the pitch and category information about the pure tone emerges right after the tone onset, and the neural code of motor response arises following the response cue frame.

### Past-trial features are reactivated by corresponding events in the current trial

Interestingly, as shown in Fig 2B, past-trial features, i.e., pitch (blue), category choice (green), and motor response (orange) could also be decoded form the neural response of the current trial. Crucially, to exclude potential current-trial confounding when decoding past-trial features, we performed the past-trial decoding analysis for each of the same current features and then combined the results (see Methods for details).

Most importantly, we found that past-trial features were reactivated by the corresponding event in the current trial (Fig 2B). Specifically, previous-pitch decoding (blue line, Fig 2B) was at chance level prior to the pure tone and rose right after tone onset (cluster-based permutation test, one-sided, corrected; significant clusters: 184 to 524 ms, 584 to 774 ms, and 954 to 1,024 ms, *p* < 0.05). Similarly, previous category choice decoding (green line, Fig 2B) emerged after the tone onset (cluster-based permutation test, one-sided, corrected, significant clusters: 84 to 154 ms, 184 to 634 ms, and 654 to 784 ms, *p* < 0.05). In contrast, rather than being triggered by the pure tone, previous motor response (orange line, Fig 2B) occurred after the response cue frame (cluster-based permutation test, one-sided, corrected; clusters: 164 to 244 ms and 264 to 604 ms after the response cue onset, *p* < 0.05). Moreover, future trial information could not be decoded (S2 Fig), and even after excluding trials preceded by f1 and f5 that belong to the unambiguous categories, past-trial pitches and category choices still showed significant reactivations in the current trial (S4D Fig).

Overall, features of the preceding trial occurring seconds before and retained in memory are reactivated by specific events in the current trial, i.e., pitch and category choice by the tone stimulus and motor response by the response cue. It is noteworthy that past-trial features, given their maintenance in memory, could potentially be reactivated by any triggering event in the current trial, yet the findings support a feature-specific reactivation temporal profile.

### Co-occurrence of past-trial reactivations and neural coding of current-trial features

We next examined the temporal relationship between the current-trial neural responses and past-trial reactivations, by calculating their cross-correlation coefficients over time. First, features in the current trial showed varied latencies in their neural response (Fig 2C). Specifically, the pitch versus category choice (green line) correlation coefficient was significant from −400 to 520 ms time lag (permutation test, one-sided, corrected, *p* < 0.05), peaking at 10 ms lag (bootstrapping, 95% CI = [0, 70] ms, *N =* 5,000), while the pitch versus motor response (brown line) showed peak around −250 ms lag (bootstrapping, *N* = 5,000, 95% CI = [−270, −230] ms; permutation test, one-sided, corrected, *p* < 0.05, − 600 to 300 ms). Thus, pitch lagged category choice by 10 ms and led motor response by 250 ms. The latter result is well expected given the 250 ms interval between pure tone and response cue frame.

Most importantly, as shown in Fig 2D, all the three features showed temporally aligned profiles between current-trial neural responses and past-trial reactivations (permutation test, one-sided, corrected, *p* < 0.05; pitch: blue, −460 to 420 ms; category choice: green, −410 to 440 ms; motor response: orange, −380 to 370 ms), peaking at 0 ms time lag (bootstrapping, *N* = 5,000, 95% CIs = [0 0] ms for pitch and category choice, and [−60, 110] ms for motor response), advocating simultaneous activation of present and past information for each feature in the present trial.

Thus, the reactivation of past-trial information occurs concurrently with the activities of present information. In other words, each feature’s current and past information arise simultaneously in the present trial, which potentially leads to the past-to-present influence and engenders serial bias.

### Past-trial feature shifts neural representation of current-trial feature

Even though past-trial reactivations co-emerge with current-trial neural responses, it remains unclear at what stage and in which direction (attractive or repulsive) past-trial information biases current neural processing. We thereby examined direct neural evidence for serial bias. Specifically, we developed a novel analysis by accessing whether the neural representation of the current-trial feature would be attracted toward or repulsed from the preceding feature.

**Fig 3:**
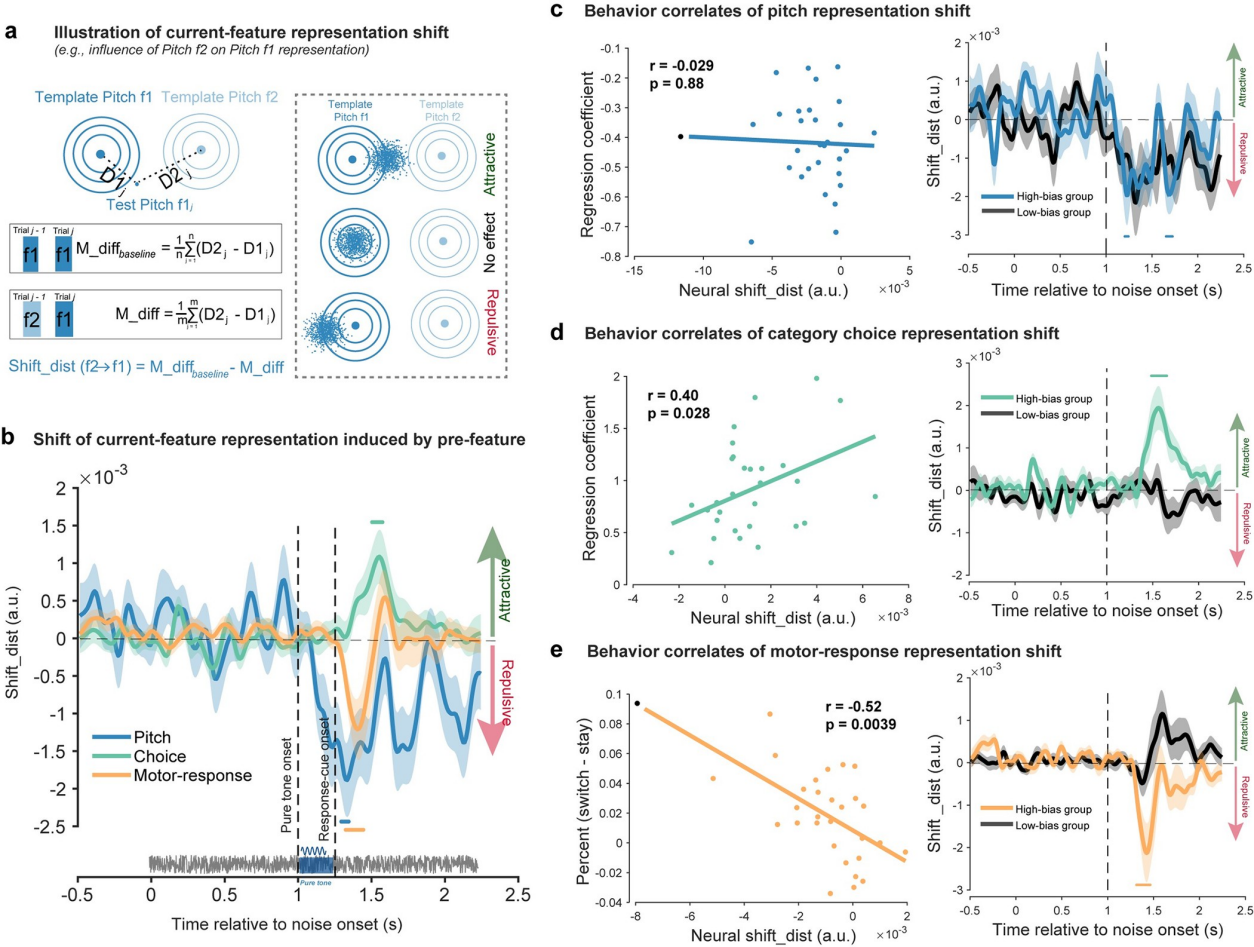
Direct neural evidence for serial bias and its behavioral relevance. **(A)** Illustration of neural representational shift analysis. Left: Influence of previous f2 on current f1 as an example. Neural templates were built for f1 (dark blue circle) and f2 (light blue circle) base on the k-fold cross validation process. For each trial with current f1 and previous f2, its neural distance to template f1 and template f2 were computed, resulting in D1, D2, and their difference M_diff (D2-D1). As baselines, trials that have current f1 but previous f1 were selected and were analyzed using the same analysis, resulting in M_diff_baseline_. The difference between M_diff_baseline_ and M_diff (Shift_dist) characterizes the neural representational shift in f1 by prior f2. Right: Neural representation of current-trial f1 is attracted toward (upper, positive Shift_dist values), repulsed from (lower, negative Shift_dist values), or not affected (middle, around zero Shift_dist values) by prior f2. (**B**) Grand average neural representational shift (Shift_dist) as a function of time following white noise onset, for pitch (blue), category choice (green), and motor response (orange). Positive and negative values correspond to attractive and repulsive direction, respectively. Horizontal colored lines denote significant temporal clusters (cluster-based permutation test, two-sided, corrected, *p* < 0.05) for each feature. Shadows represent SEM. (**C-E**) Behavioral correlates of neural shift. Left: Scatterplot of behavioral bias vs. neural shift for pitch (**C**), category choice (**D**), and motor response (**E**). Neural representation shift is the mean shift_dist across significant clusters in B. Serial bias behaviors for pitch, category choice, and motor response are indexed by regression coefficients of past pitch, past category choice, and difference between “switch” and “stay,” respectively. Each dot represents individual participant, with black dots denoting excluded participants as outliers. Right: Participants were divided into two groups based on the serial bias behavior in pitch, category choice, or motor response, respectively. Grand average neural representational shift (Shift_dist) of High-bias (colored lines) and Low-bias (black line) groups for pitch (**C**), category choice (**D**), and motor response (**E**). Horizontal colored lines denote significant temporal clusters (cluster-based permutation test, one-sided, corrected, *p* < 0.05). Data supporting this figure found here: https://osf.io/4cwv7/?view_only=3a4885189ebf46aaacf05ef109821d03.

Fig 3A (left panel) exemplifies the general idea to test pitch serial bias, e.g., quantifying the influence of past-trial f2 on current-trial f1. First, we built the neural templates for f1 (dark blue) and f2 (light blue) based on the k-fold cross validation process (see details in Methods). Next, we chose trials (current f1, previous f2) and computed the neural distance between each trial in the subsample and the f1 and f2 templates, separately, yielding D1 and D2, from which M_diff (D2-D1) was obtained. For comparison, we chose trials (current f1, previous f1) as baselines and did the same distance computation, yielding the M_diff_baseline_ (D2-D1). Finally, the difference between M_diff and M_diff_baseline_ was calculated (Shift_dist) to quantify the influence of the previous f2 on the neural representation of current f1. If previous f2 attracts f1 neural representation, we would expect smaller M_diff than M_diff_baseline_, yielding positive Shift_dist values, and vice versa. There are three possibilities—attractive, no effect, and repulsive (Fig 3A, right panel)—corresponding to positive, around zero, and negative Shift_dist values, respectively. A similar idea has been applied to category and motor response (see Methods for details; S3 Fig). The neural shift analysis was performed at each time point, for each feature, and in each participant.

Fig 3B plots the neural shift for the three features, with positive and negative values denoting attraction towards and repulsion from the past feature, separately. Pitch (blue line) showed a repulsive bias, rising shortly after pure tone (cluster-based permutation test, two-sided, corrected; 294 to 344 ms after the tone onset, *p* = 0.0056), consistent with the repulsive direction in serial bias behavior (Fig 1B and 1D). In contrast, the category choice (green line) displayed an attractive shift towards previous information, occurring relatively late (cluster-based permutation test, two-sided, corrected; 504 to 574 ms after the tone onset, *p* = 0.0058), congruent with the attractive bias in behavior (Fig 1C and 1D). Finally, the neural code of motor response (orange line) was shifted away from that of the prior trial (cluster-based permutation test, two-sided, corrected; 74 to 194 ms after the response cue, *p* = 0.005), again in line with behavior (Fig 1E). Notably, the neural shift results still held when trials preceded by unambiguous pitches (i.e., f1, f5) were removed (S4E Fig), consistent with the behavioral results (S4A, S4B and S4C Fig). Thus, the findings demonstrate direct neural evidence for serial bias, revealing a behaviorally congruent shift in neural representation for pitch, category choice, and motor response.

Together, following the reactivation of past-trial information, the current-trial neural representation shifts accordingly, in a feature-specific manner and in a behaviorally congruent way.

### Relation to serial bias behavior

Finally, by examining two neural indexes—past-trial reactivation and neural shift—we assessed the behavioral relevance of the neural signature of serial bias. First, as shown in Fig 2E, the past-trial reactivation strength, defined as the mean decoding performance within the significant temporal clusters (Fig 2B), is correlated with the corresponding serial bias behavior across participants, for pitch, category choice, and motor response (Pearson’s correlation, *N* = 30, r = −0.42, *p* = 0.022 for pitch; *N* = 29, r = 0.40, *p* = 0.033 for choice; *N* = 29, r = 0.39, *p* = 0.036 for choice). Note that the negative correlation for pitch is due to its repulsive serial bias effect. Meanwhile, as shown in Fig 3C, 3D and 3E (left), the inter-subject correlation between neural shift and behavior is only significant for category choice (Pearson’s correlation, *N =* 30, r = 0.40, *p* = 0.028) and motor response (Pearson’s correlation, *N* = 29, r = −0.52, *p* = 0.0039), but not for pitch (Pearson’s correlation, *N* = 29, r = −0.29, *p* = 0.88).

The lack of behavioral correlations could be a result of the low statistical power of pitch neural shift analysis given its 5 levels. In order to address this issue, we performed a group-division analysis to boost the signal-to-noise ratio. Specifically, for each feature, all the participants (*N =* 30) were divided into two groups of the same size—High-bias and Low-bias—based on their serial bias in behavior (see Methods for details). As shown in Fig 3C, 3D and 3E (right), the High-bias group displayed a significant neural shift for pitch (Fig 3C, blue; cluster-based permutation test, one-sided, corrected, 204 to 244 ms and 654 to 724 ms after the tone onset, *p* < 0.05), category choice (Fig 3D, green; 484 to 664 ms after the tone onset, *p* < 0.001), and motor response (Fig 3E, orange; 64 to 224 ms after the response cue onset, *p* < 0.001), while not for the Low-bias group (Fig 3C, 3D and 3E, black line).

Together, past-trial reactivation and neural shift are associated with subsequent serial bias behavior.

### Past-trial reactivations are not due to early task-relevant events or temporal prediction (Experiment 2)

**Fig 4:**
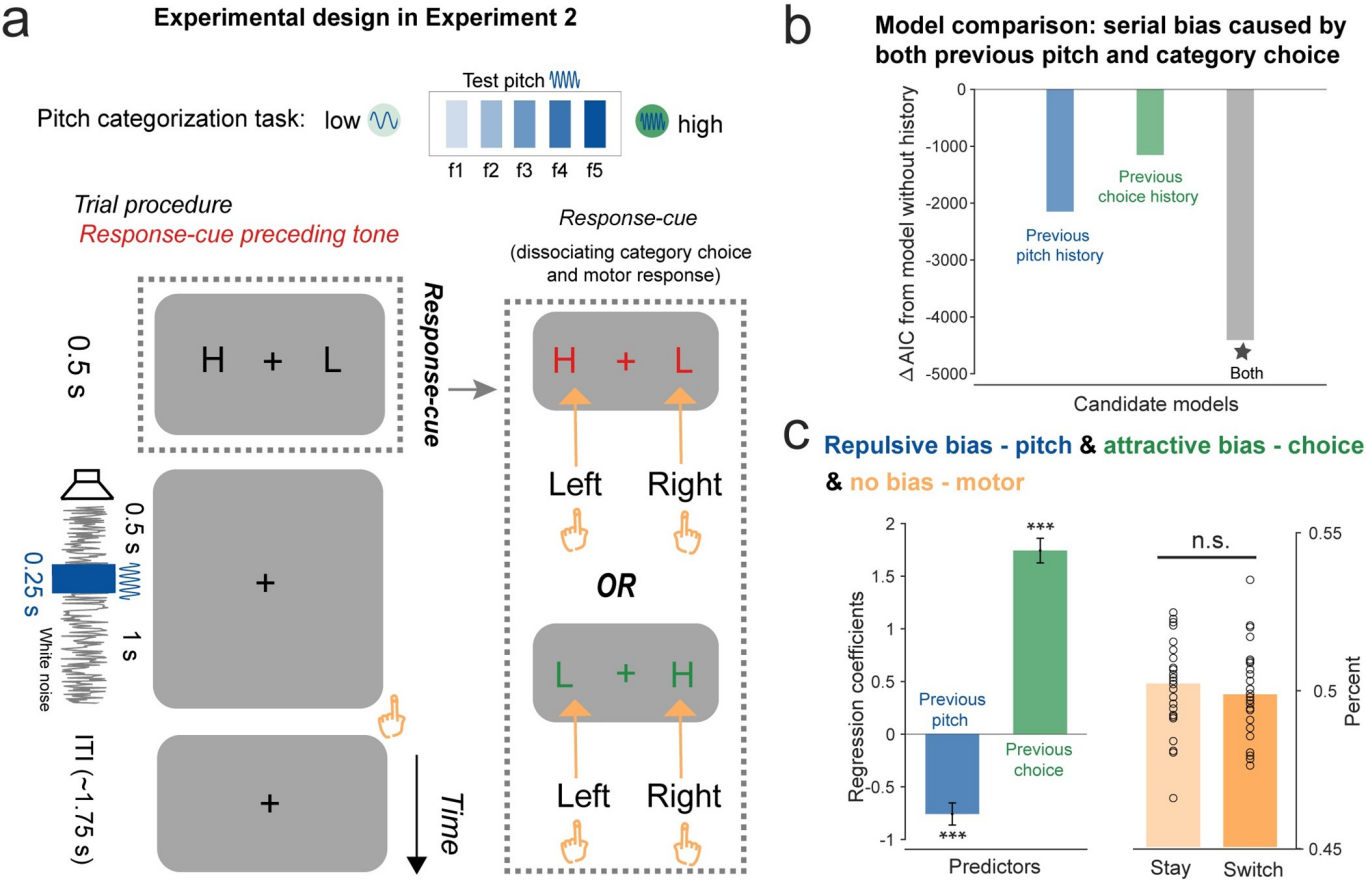
Task paradigm and behavioral serial bias in Experiment 2. (**A**) Experiment 2 employed the same auditory categorization paradigm as Experiment 1, with only one difference, i.e., the response–cue screen appeared before the white noise (dotted box). Participants categorized the following 0.25-s pure tone (dark blue) embedded in a 1.75-s sustained white noise (grey line) into “high” or “low” category, by using the corresponding hand defined in the response–cue frame (dotted box) appeared at the beginning of each trial. (**B**) Model comparison results. ΔAIC of Model 2 (blue; current trial + previous pitch), Model 3 (green; current trial + previous category choice), and Model 4 (grey; current trial + previous pitch + previous category choice), compared to Model 1 (current trial only). (**C**) Left: Regression coefficients for previous pitch (blue) and previous category choice (green) extracted from the winning model (Model 4, * in model comparison). Error bars represent 95% confidence interval. Right: No motor response serial bias, i.e., no difference between “Switch” trials (dark orange) and “Stay” trials (light orange). Each circle represents individual participant. (***: *p* < 0.001). Data supporting this figure found here: https://osf.io/4cwv7/?view_only=3a4885189ebf46aaacf05ef109821d03.

In Experiment 1, pitch and category choice information of the previous trial was reactivated by the corresponding event, i.e., pure tone (Fig 2B). Meanwhile, since the pure tone occurred early in each trial (Fig 1A), it might be the first task-relevant event (i.e., pure tone) rather than the feature-specific event that reactivated the past-trial information. To test the possibility and also confirm the findings of Experiment 1, we designed Experiment 2 (*N* = 30) during which participants performed the same auditory categorization task as Experiment 1, except now the response–cue frame appeared at the beginning of each trial (Fig 4A). If the pitch and category choice reactivations are indeed due to the early task-relevant event, we would expect their reactivations right after the response–cue frame. Moreover, since the white noise occurred at a fixed temporal lag after the response–cue frame in Experiment 2 (Fig 4A), we could also test whether the reactivation simply derives from temporal prediction.

First, participants exhibited similar serial bias behavior as Experiment 1. All the history-dependence models outperformed the history-free model (Fig 4B; Model 2 versus Model 1: ΔAIC = −2,148; Model 3 versus Model 1: ΔAIC = −1,151; Model 4 versus Model 1: ΔAIC = −4,406). Furthermore, the pitch and category choice showed negative (regression coefficient: mean = −0.76; 95% CI = [−0.96, −0.55]; t(58,196) = −7.24, *p* < 0.001, one-sample *t* test) and positive serial bias (mean = 1.74; 95% CI = [1.51, 1.97]; t(58,196) = 14.90, *p* < 0.001, one-sample *t* test), respectively (Fig 4C, left). Meanwhile, Experiment 2 did not show serial bias for motor response (Fig 4C right, “Switch”: mean = 0.50, SD = 0.014; “Stay”: mean = 0.50, SD = 0.014; “Switch” versus “Stay”: paired-sample *t* test, t(29) = −0.66, *p* = 0.52), consistent with previous results [11].

**Fig 5:**
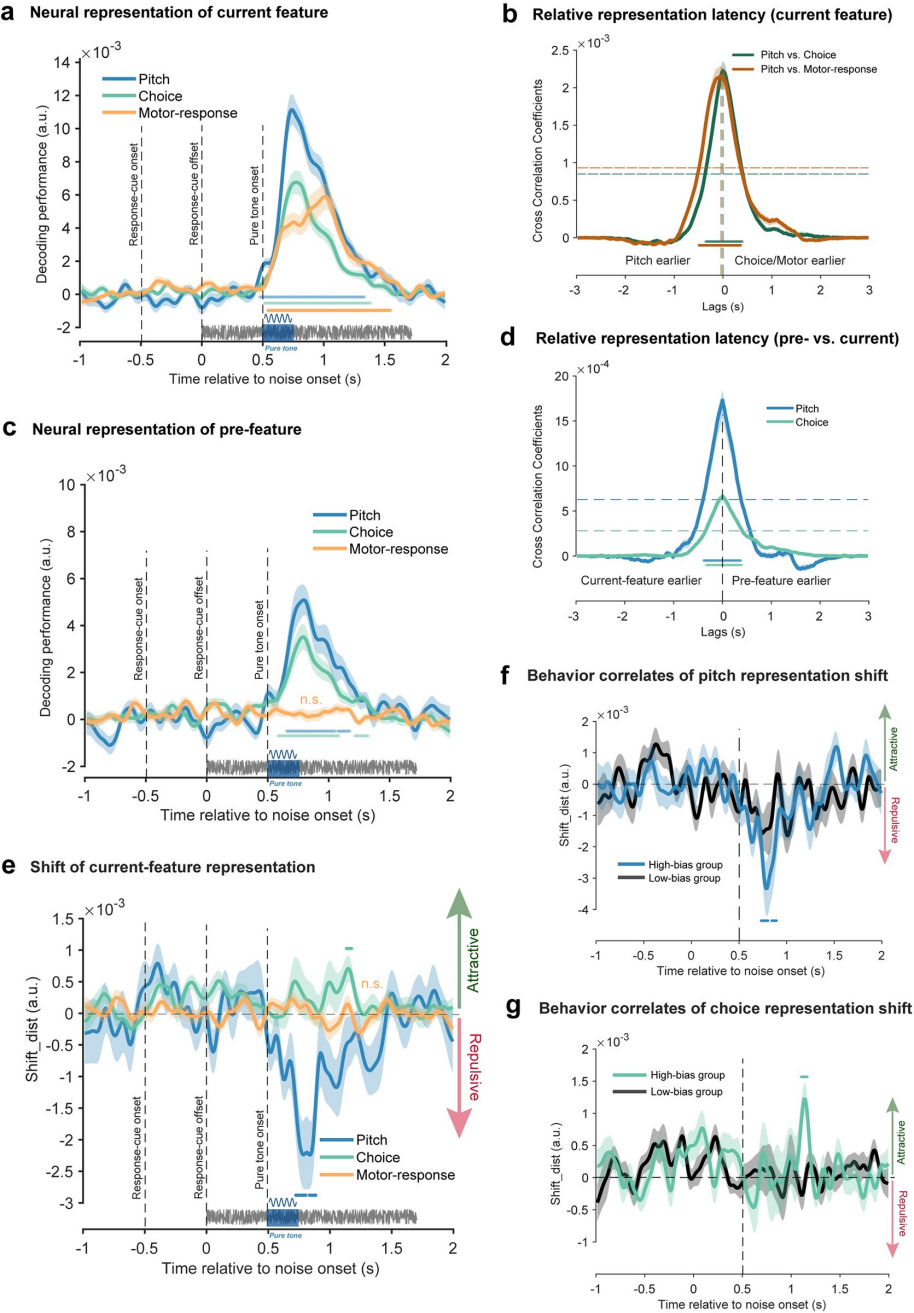
Neural results of Experiment 2. **(A)** Grand average decoding performance for current-trial features as a function of time following the sustained white noise, for pitch (blue), category choice (green), and motor response (orange). Note that the response–cue frame occurred before white noise onset (−0.5 s, dashed vertical line). Time 0 denotes the white noise onset. Vertical dashed lines from left to right denote the onset and offset of response–cue frame, and the pure tone onset, respectively. Horizontal colored lines denote significant temporal clusters (cluster-based permutation test, *p* < 0.05, one-sided, corrected) for each feature. Shadows represent SEM. (**B**) Cross-correlation coefficient time course as a function of temporal lag, for each current-trial feature-pair (green: pitch vs. category choice; brown: pitch vs. motor response). Shadow represents 95% confidence interval (bootstrapping, *N* = 5,000). Horizontal colored lines denote significant temporal clusters (permutation test, one-sided, corrected, *p* < 0.05) for each feature pair. Horizontal dashed lines represent the 95% permutation threshold (corrected for multiple comparison). Vertical dashed line indicates the peak correlation coefficient indexing the relative activation lag. (**C**) The same as A but for past-trial features. (**D**) Cross-correlation coefficients between past-trial reactivation and current-trial neural response, as a function of temporal lag, for pitch (blue) and category choice (green). The peak at 0 ms temporal lag (vertical dashed line) supports their co-occurrence. Other denotations are the same as B. (**E**) Grand average neural representational shift (Shift_dist) as a function of time relative to the white noise onset, for pitch (blue), category choice (green), and motor response (orange). Positive and negative values correspond to attractive and repulsive direction, respectively. Horizontal colored lines denote significant temporal clusters (cluster-based permutation test, one-sided, corrected, *p* < 0.05 for pitch, *p* = 0.056 for category) for each feature. Shadows represent SEM. (**F, G**) Participants were divided into two groups based on the serial bias behavior in pitch and category choice, respectively. Grand average Shift_dist of High-bias (colored lines) and Low-bias (black line) groups for pitch (**F**) and category choice (**G**). Horizontal colored lines denote significant temporal clusters (cluster-based permutation test, one-sided, corrected, *p* < 0.05). Data supporting this figure found here: https://osf.io/4cwv7/?view_only=3a4885189ebf46aaacf05ef109821d03.

Importantly, as shown in Fig 5, Experiment 2 did not show an early reactivation of past-trial information after the response–cue frame or the white noise but, instead, largely replicated Experiment 1. First, neural representation of the current-trial features emerged after the tone stimulus (Fig 5A, cluster-based permutation test, *p* < 0.001, one-sided, corrected; −26 to 834 ms for pitch; 14 to 884 ms for category choice; 44 to 1,044 ms for motor response), with similar latencies (Fig 5B; pitch versus category choice: correlation coefficients, 0 ms lag, bootstrapping, 95% CI = [−10, 20] ms, *N =* 5,000; pitch versus category choice: permutation test, one-sided, corrected, −360 to 400 ms; pitch versus motor: correlation coefficients, −40 ms lag, bootstrapping, *N* = 5,000; pitch versus motor: 95% CI = [−150, 0] ms, permutation test, one-sided, corrected, *p* < 0.05, − 490 to 360 ms). Second, past-trial pitch (blue) and category choice (orange) features could also be decoded from neural response (Fig 5C; cluster-based permutation test, one-sided, corrected; pitch: 154 to 554 ms and 574 to 674 ms after tone onset, *p* < 0.05; category: 84 to 584 ms and 714 to 824 ms after tone onset, *p* < 0.040). Critically, similar to the findings of Experiment 1, past-trial reactivations co-occurred with the current-trial neural response of features, for both pitch and category choice (Fig 5D; cross-correlation coefficients: 0 ms lag for both pitch and category choice, bootstrapping, 95% CIs = [0, 0] ms, *N* = 5,000; permutation test, one-sided, corrected, pitch: −390 to 380 ms, category choice: −340 ms to 400 ms).

Finally, Experiment 2 also replicated the behaviorally relevant neural shifting results, for both pitch and category choice (Fig 5E). Specifically, neural representations of the current-trial pitch (blue) and category choice (green) displayed repulsive and attractive shifts by previous features, respectively (cluster-based permutation test, one-sided, corrected; pitch: 224 to 314 ms and 334 to 384 ms after the tone onset, *p* < 0.01; category choice: 634 to 674 ms after the tone onset, *p* = 0.056). Moreover, the neural shifts were correlated with serial bias behavior for both pitch (Fig 5F) and category choice (Fig 5G), i.e., showing significant neural shifts for the High-bias but not Low-bias group (cluster-based permutation test, one-sided, corrected; pitch: 234 to 304 ms and 344 to 394 ms after tone onset, *p* < 0.05; category: 594 to 674 ms after tone onset, *p* = 0.017). Notably, consistent with the lack of behavioral serial bias for motor response (Fig 4C), we found neither significant past-trial reactivation (Fig 5C, orange) nor neural shift for motor response (Fig 5E, orange).

There are also some incongruencies between Experiments 1 and 2. First, motor response emerges simultaneously with pitch and choice, suggesting that instead of being an output stage, the motor system actively participates in decision-making at the early stage [11,42]. In addition, past-trial reactivation and the corresponding behavioral bias were not observed in Experiment 2. One possible explanation is that early planning of choice-motor mapping could disrupt latent memory of previous motor response traces.

Together, past-trial features could not be reactivated by either an early task-relevant event (i.e., response–cue frame) or a temporally predictable stimulus (i.e., white noise). Instead, only the corresponding feature-specific event could trigger the past-trial reactivations, with their co-occurrences giving rise to serial bias.

## Discussion

In two auditory categorization experiments, we reveal a new feature-specific reactivation neural mechanism for serial bias that occurs spontaneously across trials. Specifically, serial bias arises from the co-emergence of past reactivation and the neural coding of present inputs, which renders their interactions and the shifting of current information. Importantly, we provide converging behavioral and new neural evidence that the serial bias occurs in a feature-dissociated manner, i.e., sensorimotor (i.e., pitch and motor response) and abstract (i.e., category choice) features are repulsed from and attracted to the corresponding ones in the previous trial, respectively, within the same task. Moreover, the past-trial reactivation is not due to either early task-relevant events or temporal prediction but triggered by the occurrence of the feature-specific event. Taken together, features in the current trial automatically trigger the corresponding memory traces of past trials, and their co-occurrence leads to serial bias. This reactivation might reflect a fundamental operational mechanism for past-to-present adaptive optimization.

The past-to-present influences occur in a wide range of paradigms and contexts [2,43,44], of which the serial bias represents an extreme phenomenon. It refers to the involuntary, systematic shifting of current perception by previous trials and appears on many features [3,4,6–10,15,45]. The typical serial dependence effect is in an attractive direction [3,10,15,45], while the repulsive bias and individual difference have also been observed [7,8,46,47]. Here, by carefully disentangling features in the experimental design, we revealed, within the same task, the dissociated serial bias for pitch, category, and motor response. We found that pitch and motor response were repulsed from past information, while the category displayed an attractive direction.

What accounts for the dissociated bias direction for pitch and category choice? We postulate that the repulsive bias for pitch reflects a sensory adaptation process. First, the repulsive bias occurs not just for unambiguous pitches in the previous trial (f1, f5), wherein participants might have a reverse expectation (e.g., after “low” categorical choice for f1, they expect the next to be a “high” category), but also for pitches associated with ambiguous categories (i.e., f2, f3, f4). Second, the neural shift of pitch occurs at a relatively early latency, indicating its sensory nature, consistent with previous findings [37,38]. About the attractive serial bias for category choice, there are currently two major views. One posits that both attractive and repulsive serial biases arise at the perceptual stage [15–18], while the two-stage framework attributes the repulsive and attractive serial bias to perceptual and post-perceptual stages, respectively [5,8,14,38,48], by incorporating efficient coding and Bayesian inference. Our results could not distinguish the two views, since the EEG recordings lack enough resolution to localize the involved brain regions. Future studies are needed to examine the spatiotemporal neural correlates of the attractive serial bias.

For the past to influence the present, either voluntarily or spontaneously, prior information should leave traces in memory. It has long been viewed that the WM process relies on persistent firing [49–52] or frequency-specific neural oscillations [53,54]. Interestingly, information could also be retained in an “activity-silent” way [21–25]. However, the “activity-silent” mode only passively maintains information [22] (including ours; see [55–57]), and memory manipulation still relies on the reactivations of WM to active states [28,29]. Consistent with the view, we demonstrate the co-emergence of past reactivation and present information, followed by the shifted neural representation of current features. Moreover, the reactivation profiles encompass multiple feature-specific reactivations that arise simultaneously as the corresponding features in the current trial. Thus, the memory traces are reactivated by corresponding features in the current trial, and the feature-specific co-occurrence in neural space contributes to the serial bias.

A recent interesting study revealed brief reactivation of past spatial information during the intertrial interval (ITI) [33], while here we observed the concurrent emergence of past reactivation and present information after the stimulus, as also revealed in other EEG findings [30–32]. Both findings support past reactivation from an activity-silent to an activity-based state for serial bias to occur, but at different time points, i.e., prestimulus versus poststimulus. The difference might arise from temporal anticipation in the task design, with a fixed ITI in the previous study but a randomized ITI in our experiment. Indeed, it is found that alpha-band power carries past-trial information only when the stimulus is temporally predictable [33]. Importantly, our Experiment 2 excludes the temporal expectancy interpretation, since even though the white noise appears at a fixed time after the response–cue frame, pitch reactivations are still triggered by the auditory tone rather than the white noise.

Due to the different tasks implemented here, we have opted to decode broad-band neural signals rather than specific neural oscillations. Alpha-band power has been mainly found in spatial WM tasks (e.g., [33,53,54,58]), while here participants performed an auditory categorization task entailing no spatial memory or attention. Additionally, we aimed to find the neural signature of multiple features, so focusing on broad-band signals rather than specific neural rhythms would be a more conservative choice. In fact, broad-band decoding has been widely used in a series of WM studies (e.g., [22,27,56,57]) and serial dependence works [30–32].

Partially consistent with previous findings [33], our alpha-band analysis during the prestimulus period revealed reactivations of past choice and motor response, but not pitch information (S5 Fig). Given the fixed time interval between noise and tone, our results indicate that the temporal anticipation of response-related features (choice and motor response) reactivates the corresponding information within the alpha-band. Moreover, persistent oscillatory activities have been found to convey past choice and motor information [11,12]. It is therefore also possible that prestimulus reactivation carried by alpha-band power reflects an enhancement of weakly but actively coded memory traces via an anticipation-induced decrease in cross-trial variability [59]. Nevertheless, reactivation on its own, either before or after stimulus, could not fully characterize the dynamic operation of serial bias. This is because reactivation only signals the presence of past-trial information, but how the past influences the present, as shown by serial bias behavior, still cannot be well accounted for. Furthermore, computational models postulate an integration of activity-based and activity-silent mechanisms for serial bias, i.e., activity-silent synaptic plasticity leads to serial bias that can be enhanced by past reactivation in WM tasks [33–35]. Meanwhile, they could not account for multifeature serial biases in non-WM tasks. Here, we provide direct neural evidence that current information is indeed shifted towards or away from the past (i.e., attractive, repulsive) following their co-occurrence.

Importantly, we extend previous single-feature emphasis to a more general framework, wherein multiple features and their respective serial biases are disentangled in both behavior and neural representations. It is recognized that even the simplest perceptual decision task encloses packs of features, e.g., physical properties, abstract categories, and response actions, collectively constituting an “event-file” [60]. Here within the same auditory categorization task, the past “event-file” is implicitly imprinted in memory and automatically passed to the next “event-trial” in a feature-encapsulated way. Importantly, not any events can trigger past information, also in line with previous findings [56,57]. For instance, white noise, despite being an auditory sound within the same sensory modality, failed to reactivate prior pitch information, and previous motor response, although apparently retained in memory, could not be triggered by either white noise or tone stimulus. Moreover, the fact that white noise reactivates memorized pitch [27,56] during maintenance but not here indicates different mechanisms for WM and serial bias. While WM task involves explicitly storing information in WM, serial bias automatically occurs without requiring participant to voluntarily retain past-trial information. The findings that white noise could not reactivate past-trial information but transcranial magnetic stimulation (TMS) could [33,61] are in line with the interpretation that white noise impulses do not reactivate synaptic traces [59]. Overall, past trial automatically leaves a memory trace within which multiple features keep their identities and specificities, exerting their respective impacts on the future.

Serial bias denotes a dramatic case about past-to-present influence since information in the previous trial, which occurs several seconds ago and in principle should be discarded, still biases the present trial. The phenomenon is therefore independent of several factors involved in many other paradigms, such as voluntary attention, task-relevant modulation, task-irrelevant capture, etc. [22,62,63]. Moreover, as trials are typically several seconds apart with random ITI in-between, serial bias could not arise from within-trial temporal effects either [43,44]. Furthermore, while potentially sharing similar WM storage underpinnings, serial bias essentially differs from WM studies that would instruct participants to at least retain certain information. Our findings thus implicate a presumably ubiquitous mechanism for temporal dependence in many cognitive processes, such as perception, attention, memory, and decision-making.

Taken together, every single present is intertwined with the past, yet the past-to-present influence is not feature-agnostic but feature-specific, allowing for information encapsulation through trial-by-trial updates. The current feature reactivates the corresponding traces left in memory, and their co-occurrence induces the neural interactions and generate serial bias, thereby facilitating automatic adaptive generalizations from past to present.

## Methods

### Experiment procedures and participants

#### Participants

Thirty participants (20.8 ± 2.2 years old, 12 females) took part in Experiment 1, and 30 new participants (20.6 ± 1.7 years old, 14 females) participated in Experiment 2. The sample size was determined based on previous serial dependence behavioral studies [7]. They are all right-handed with normal or corrected to normal vision and normal audition. Participants were paid and gave written informed consent before starting the experiment. The study was conducted in accordance with the Declaration of Helsinki and was approved by the Ethical Committee of the School of Psychological and Cognitive Sciences at Peking University.

#### Apparatus

Experimental programs were developed using Matlab (MathWorks, Natick, MA, USA) and Psychophysics Toolbox [64]. Auditory stimuli were controlled by external sound card RME Babyface pro and were emitted through Sennheiser CX213 earphones. The loudness of sound was adjusted to a comfortable level (approximately 65 dB SPL). The visual stimuli were presented on a Display++ LCD screen with a refreshing rate of 120 Hz and a resolution of 1,280 × 1,024 pixels. Participants sat at a distance of 100 cm from the screen and their heads were maintained steady using a chin rest. In the main task, a Cedrus RB-540 response box was used to collect participants’ responses.

#### Stimuli and experimental procedure

The experiment consisted of two phases, a behavioral training phase and a formal testing phase. In the behavioral training phase, participants had to memorize two tones, a “low” tone (180 Hz) and a “high” tone (360 Hz), without EEG recordings, so that the “low-pitch” and “high-pitch” categories could be successfully formed before the formal test. In the formal testing phase, participants performed a categorization task, reporting whether a given auditory tone is more similar to the memorized “low” tone or “high” tone, i.e., an auditory categorization task, with EEG recordings.

#### Behavioral training phase

We designed two types of tasks to help participants memorize these two tones (see S1 Fig): a reproduction task and a recall task. In the reproduction task, on each trial, the pure tone (250 ms, 10 ms ramp-up, ramp-down time) was presented first and participants were required to reproduce it afterward. In the recall task, a recall cue (“low” or “high”) was presented to participants at the beginning of each trial. They were asked to produce it according to their memory. The training phase consisted of two sessions. The first session is a block-wise design with four 10-trial blocks containing a “high” tone reproduction task, “high” tone recall task, “low” tone reproduction task, and “low” tone recall task, respectively. The order of “high” or “low” tones is counterbalanced among participants. In the second session, the stimuli were trial-by-trial randomized. This session had two 40-trial blocks, with the first block containing a reproduction task with feedback and the second block containing a recall task without feedback.

For the reproduction task, participants were presented with a 250-ms pure tone (180 Hz or 360 Hz), and 3 s later, they adjusted the pitch of a probe tone, which was randomly selected from 13 initial frequencies (mean is the probe frequency, log step 0.1) with up and down arrows (log step 0.1) to match the frequency to the target tone. For the recall task, a retro-cue (“high” or “low”) was presented for 2 s. Participants then used up and down arrows to adjust the tone frequency to match the target tone (180 Hz or 360 Hz). The ITI was 1 to 1.5 s. Participants moved to the testing phase only when they accomplished the training phase tasks.

Participants with poor recalling performance in the training session would be excluded from participating in the formal test.

#### Formal testing phase

Participants had to compare the probe tone to the memorized “low” tone (180 Hz) and “high” tone (360 Hz) and chose whether the tone was more similar to the “low” or “high” tone. To equalize the task difficulty across participants, we individualized the probe tones. First, we used the same five probe tones (202, 227, 255, 286, and 321 Hz) to test each participant’s performance. Then, we fitted a logistic regression to the percentage of “high” choices as a function of pitches and acquired pitches (f1, f2, f3, f4, f5) corresponding to percents 0.1, 0.3, 0.5, 0.7, and 0.9 for each participant. Then, we employed these five pitches in the main task when EEG signals were recorded.

***Experiment 1*** In each trial, the probe tone embedded in white noise was presented while participants kept their eyes on the central cross. The added white noise is to prime the auditory system so that the subsequent pitch decoding analysis would not be dominated by auditory onset response. The white noise lasted 2.25 s, and the probe tone (duration, 0.25 s) was presented 1 s after noise onset. After the offset of the probe tone, a response cue was immediately presented on the screen, based on which participants chose the corresponding hand to make a response. For example, if the “high” and “low” characters were presented on the left and right side, respectively, participants had to press a left button for “high” and a right button for “low” categorical choice, and vice versa. Participants were required to respond as accurately and fast as possible. The ITI is 1.5 s to 2 s. The formal test consisted 20 blocks with 100 trials for each block. Five tones (f1, f2, f3, f4, and f5) were pseudorandomized within each block (20 trials for each pitch). Participants performed 2,000 trials in total, which were divided into two sessions accomplished on two different days.

***Experiment 2*** The stimuli and task were similar to those in Experiment 1, except that the response cue was presented before the tone onset and that the white noise lasted 1.75 s and the probe tone (duration, 0.25 s) appeared 0.5 s after noise onset. Specifically, at the beginning of each trial, a response cue was presented for 0.5 s, which was followed by the auditory stimuli. Participants chose the corresponding hand to make a response based on the response cue. The response cue was denoted with letters on screen and colors (red or green).

### Behavioral data analysis and modeling

#### Aggregate analysis

To examine the pitch serial bias, i.e., how current categorical decisions are influenced by pitches in the previous trial, we first combined data from all participants into aggregated data. The aggregated data were then divided into five groups based on the previous pitch, and then for each group, we calculated the percentage of “high” choices for each pitch on the current trial. Similarly, to examine the serial bias for category choices, i.e., how current categorical decisions are influenced by category choices on the previous trial, data were divided into two groups based on the previous category choice. To examine whether the previous-current stimulus distance can modulate the serial bias in category, for each of the 25 conditions (5 previous pitches × 5 current pitches), we calculated the “high” choice percentage difference between trials preceded by the “high” choice and “low” choice and then averaged according to the stimulus distance. Further permutation test (*N* = 5,000, null distribution created by shuffling the trial order) and bootstrapping (*N* = 5,000) were performed to assess significance and confidence intervals of the effects. Finally, we also examined whether there is a serial bias in motor response by comparing the switching rate (percentage of trials with motor responses different from the previous response) and staying rate (1-switching rate).

#### GLM models

To visualize the overall pattern of serial bias, for each group of the aggregate data, we first fitted a logistic regression model (glmfit function in the Matlab Statistics and Machine Learning Toolbox) to characterize the relationship between the percentage of “high” choices and the pitch on the current trial.

To quantitatively test the serial bias caused by previous pitch and its corresponding category, we built four GLMMs with Model 1 assuming no history influence, Models 2 and 3 assuming previous pitch and previous category affect current decisions, respectively, and Model 4 assuming both previous pitch and category information influences current decision-making. The mixed-effect models account for group-level fixed effects and random effects due to variation across participants. The categorical response was modeled using a Bernoulli distribution, *Y*_*ij*_~*Bernoulli*(*p*_*ij*_), and the full model (model 4) is given by:

$$p_{ij}={f(\beta }_{0i}+\beta_{1i}{CurrentPitch}_{ij}+\beta_{2i}{PrePitch}_{ij}+\beta_{3i}{PreCategory}_{ij})$$ (1)

$$\beta_{0i}=\gamma_{0}+\mu_{0i};\;\beta_{1i}=\gamma_{1}+\mu_{1i};\;\beta_{2i}=\gamma_{2}+\mu_{2i};\;\beta_{3i}=\gamma_{3}+\mu_{3i}$$ (2)

where *i* indicates participant index and *j* represents the *jth* trial, and $f(x)=\frac{e^{x}}{1+e^{x}}$ indicates the logistic link function. *Y*_*ij*_ is the categorical choice (1 for “high”, 0 for “low”) in the *jth* trial for participant *i*. Parameters *γ*_0_, *γ*_1_, *γ*_2_, *γ*_3_ denote the group-level regression coefficients (fixed effect) for intercept, predictor current pitch, predictor previous pitch, and predictor previous category, respectively. The vector μ = (*μ*_0*i*_, *μ*_1*i*_, *μ*_2*i*_, *μ*_3*i*_)^T^ denotes the corresponding individual-level random effect with μ∼N(0,Σ) where Σ is the covariance matrix.

The generalized mixed-effects models were performed using *fitglme* function (Distribution: Binomial, Link: logit) in the Matlab Statistics and Machine Learning Toolbox. Model performance was evaluated using the AIC with a smaller value indicating better performance. Specifically, we calculated the *ΔAIC* of Models 2 to 4 from the AIC of the model without history influence (Model 1).

### EEG data analysis

#### EEG acquisition and preprocessing

The EEG signals were acquired using a 64-electrode actiCAP system and two BrainAmp amplifiers through BrainVision Recorder software (Brain Products). One electrode placed below the right eye was used to record the vertical electrooculography. The impedance of all electrodes was kept below 10 kΩ. During data acquisition, the signals were referenced to electrode FCz and sampled at 500 Hz. We analyzed the data using Matlab custom codes and the FieldTrip toolbox. Data were epoched 0.5 s before noise onset and 2.25 s after noise onset in Experiment 1, and 0.5 s before response cue and 2 s after noise onset in Experiment 2. The epoched EEG signals were referenced to the mean signal across all electrodes and baseline correction was performed using signals from −0.5 s to −0.3 s relative to noise onset for Experiment 1 and no baseline correction was performed in Experiment 2. Then, signals were bandpass filtered between 1 and 30 Hz and down-sampled to 100 Hz. Ocular and other artifacts were removed from the data using independent component analysis (ICA). Guided by visual inspection, epochs with excessive variance were manually excluded from the following analysis. Epochs with reaction times exceeding four standard deviations were also excluded. On average, 40 ± 10 trials in Experiment 1 and 40 ± 12 trials in Experiment 2 were excluded.

#### Multivariate pattern analysis

We employed a time-resolved multivariate decoding method [22,39], i.e., the Representational Similarity Analysis (RSA) [40] with Mahalanobis distance [41] to examine the neural representation of different features (pitch, category, and motor response) across time. At each time point, the neural information linked to each feature was decoded by taking advantage of spatial–temporal voltage dynamics in a predefined time window (20 ms) to increase decoding accuracy. Specifically, at a given time point t, the voltage fluctuations over space (i.e., 64 electrodes) and time (t and t-1) were pooled together as a multivariate pattern (128 values). Using 8-fold cross-validation, the Mahalanobis distance between each of the left-out trials and the mean of condition-specific train trials was then calculated with the covariance matrix estimated from the train trials using a shrinkage estimator [65]. Notably, for the seven train-folds, the number of trials for each condition of a certain feature (e.g., pitch) was equalized by randomly subsampling the minimum number of condition-specific trials among different conditions so that the training set would not be biased.

After obtaining the neural representation similarity (Mahalanobis distance) between paired conditions of a certain feature for each trial, we further performed linear regression to quantify the relationship between neural representation similarity and feature similarity. Here, the predictor, feature similarity was dummy coded (0 for pair of the same condition, 1 for pair of different conditions). The averaged regression coefficient of all the trials was used to represent the decoding performance. To obtain reliable estimates of decoding performance, we repeated this procedure 50 times in each of which we randomly divided the dataset into 8 folds.

With the same EEG signals, we also computed the decoding performance for each of the current-trial and previous-trial features (pitch, category, and motor responses) independently. Importantly, to exclude potential interference between current and previous features during decoding, we decoded one by fixing the other. For example, when we decoded the pitch information on the current trial, we first divided the trials into five subsets based on the pitches in the previous trials. Then, we computed the decoding performance in each subset and then averaged them. Similarly, when decoding the previous-trial pitch, we performed the analysis on trials containing the same current-trial pitch, respectively, and then averaged the results.

#### Statistical analysis

We used a nonparametric sign-permutation test to examine whether the decoding performance was significantly larger than 0 at the group level [66]. For each permutation, the sign of each participant’s decoding performance was randomly flipped with a probability of 50% and the group mean of decoding performance was computed. We conducted permutation 100,000 times and created the null distribution. The group means of original decoding performance were compared against the null distribution, and the *p*-value was calculated as the percentage of values in the null distribution that is larger than the real mean. This permutation test was performed for each time point of the whole time course. Then, a cluster-based permutation (*N* = 100,000) was used for multiple comparisons across time in which cluster was defined based on the threshold of *p* = 0.05. The statistical analysis was performed on the raw data of decoding performance, and we smoothed the group means of decoding time course using a Gaussian-weighted moving average filter with a 150-ms time window to better visualize the result when plotting.

#### Cross-correlation analysis

To examine the relative temporal lags of neural representations of different features, we performed cross-correlation analysis for different pairs of decoding performance time series. First, we tested whether the time course differs for different features in the current trial (pitch versus category, pitch versus motor response). Next, we investigated whether there was a time lag between the representation of the same feature in previous and current trials. Specifically, for each paired time series (e.g., current pitch versus current category), the cross-correlation (xcorr function in Matlab) between their group mean time courses were computed as a function of time lags between the time series. Then, we used a nonparametric permutation test [67] to test its significance. For each permutation, each of the two group mean time courses was shuffled across time following which the cross-correlation was calculated, and then the maximum of the correlation coefficients was selected (multiple comparison corrected). This procedure was conducted 100,000 times to create the null distribution. The permutation test was run to assess whether the original cross-correlation coefficient was higher than the 95% of the values from the null distribution.

#### Neural representation shift analysis

To directly examine the neural representation of serial bias for each feature, we developed a new test to access whether and how the previous-trial feature affects the neural representation of the current-trial feature and in which direction, i.e., attractive or repulsive.

Generally speaking, for each feature (pitch, category, and motor response), we built neural templates by averaging the condition-specific trials and then calculated the Mahalanobis distance between each trial and the templates. Specifically, we employed the same 8-fold cross-validation as in the multivariate pattern analysis, i.e., first building neural templates by averaging the seven train-folds trials, followed by calculating the Mahalanobis distance between each of the left-out trials and the templates, for each 8-fold division. After repeating this procedure 50 times, we obtained the reliable estimates of neural distance to the templates for each trial. Moreover, considering the strong motor signals in Experiment 1 (Fig 2A), the motor response was regressed out when performing pitch decoding analysis, to increase the decoding sensitivity. In addition, given the temporal binding between motor response and category in Experiment 2 design (Fig 5A and 5B), the response cue was controlled when performing category decoding analysis. We next analyzed whether and how the previous-trial feature influences the neural representation of feature in the current trial.

For pitch neural shift analysis, we took an example of quantifying the influence of past-trial f2 on current-trial f1 for illustration of the general idea (see Fig 3A), as illustrations. First, the neural templates for f1 (dark blue) and f2 (light blue) were selected. Next, we chose trials (current f1, previous f2) and computed the neural distance between each trial in the subsample and the f1 and f2 templates, separately, yielding D1 and D2, from which M_diff (D2-D1) was obtained. For comparison, we chose trials (current f1, previous f1) as baselines and did the same distance computation, yielding the M_diff_baseline_ (D2-D1). Finally, the difference between M_diff and M_diff_baseline_ was calculated (Shift_dist) to quantify the influence of the previous f2 on the neural representation of current f1. If previous f2 attracts f1 neural representation, we would expect smaller M_diff than M_diff_baseline_, yielding positive Shift_dist values, and vice versa. There are three possibilities—attractive, no effect, and repulsive (Fig 3A, right panel)—corresponding to positive, around zero, and negative Shift_dist values, respectively. The neural shift analysis was performed at each time point, for each participant.

For category neural shift analysis (see S3 Fig), we first built the neural templates for the “high-pitch” (dark green circle) and “low-pitch” (light green circle) categories, based on all trials (approximated by 8-fold cross validation as the above pitch shift analysis). Next, we computed the neural distance between each trial and the “high” and “low” templates, yielding D1 and D2, respectively. Positive serial bias would predict neural attraction to the past-trial category, i.e., positive Diff_High (D1-D2) or positive Diff_Low (D2-D1) values when preceded by the “high” and “low” category, correspondingly. We then averaged the Diff-High and Diff_low values as Shift_dist to characterize the neural shift of category information. There are three possibilities—attractive, no effect, and repulsive—corresponding to positive, around zero, and negative Shift_dist values, respectively. The neural shift analysis was performed at each time point and in each participant.

A similar idea as category analysis has been applied to motor response. Specifically, we first built the neural templates for the “Left” and “Right” motor responses, based on all trials. Next, we computed the neural distance between each trial and the “Left” and “Right” templates, yielding D1 and D2, respectively. Positive serial bias would predict neural attraction to the past-trial category, i.e., positive Diff_High (D1-D2) or positive Diff_Low (D2-D1) values when preceded by the “Left” and "Right" categories, correspondingly. We then averaged the Diff-High and Diff_low values as Shift_dist to characterize the neural shift of category information. There are three possibilities—attractive, no effect, and repulsive—corresponding to positive, around zero, and negative Shift_dist values, respectively. The neural shift analysis was performed at each time point and in each participant.

A similar nonparametric sign-permutation test and cluster-based multiple comparison correction were performed on the neural shift analysis results. Finally, to examine the behavioral relevance of the neural shifts, for each feature, we divided participants into two groups (High-bias versus Low-bias) based on behavioral serial bias (the regression coefficient extracted from the GLM Model 4) and then performed the same neural shift analysis for the two groups, respectively.

## Supporting information

S1 Fig Pretest task paradigm and behavioral performance. (**A**) Tone memory task paradigm. Upper: A reproduction task where participants were required to reproduce the given tone (180 or 360 Hz) by using the up and down arrow keys, after which feedbacks were provided. Lower: A recall task where participants had to recall the respective tone according to the retro-cue “high” (360 Hz) or “low” (180 Hz) and reproduce it. The pretest contains a block-design session (two reproduction blocks and two recall blocks each of which testing only one tone for 10 trials) and a randomization design session (one reproduction block and one recall block each of which testing two tones in a trial-by-trial random manner, 20 trials for each tone). (**B, C**) Recall performance in the randomization design session of Experiments 1 (**B**) and 2 (**C**). Both plots show two clear peaks around 180 Hz and 360 Hz (aggregate results across participants). Data supporting this figure found here: https://osf.io/4cwv7/?view_only=3a4885189ebf46aaacf05ef109821d03.
(TIF)

S2 Fig Decoding performance of past-trial and future-trial features. Grand average decoding performance for previous features (in color) and future features (in black) as a function of time following white noise onset, for pitch (left), category choice (middle), and motor response (right). The vertical dashed lines denote the tone onset and the response–cue onset. Horizontal colored lines denote significant temporal clusters (cluster-based permutation test, two-sided, corrected, *p* < 0.05) for each past feature, and the horizontal black line in the middle panel indicates a marginal significant temporal cluster (cluster-based permutation test, two-sided, corrected, *p* = 0.056). Shadows represent SEM. Data supporting this figure found here: https://osf.io/4cwv7/?view_only=3a4885189ebf46aaacf05ef109821d03.
(TIF)

S3 Fig Illustration of neural representational shift analysis (category choice as an example). **Left**: Two neural templates were built for “high” (dark green circle) and “low” (light green circle) category choice base on all trials. For each trial, its neural distance to the two templates were computed, resulting in D1 and D2, respectively. Positive serial bias would predict neural attraction to previous category choice, i.e., positive Diff_High (D2-D1) and positive Diff_Low (D1-D2) values when preceded by “high” and “low” category choice, respectively. The two values were averaged as Shift_dist to characterize the neural shift for category choice. **Right**: Neural representation of current-trial category is attracted toward (upper, positive Shift_dist values), repulsed from (lower, negative Shift_dist values), or not affected (middle, around zero Shift_dist values) by prior category information.
(TIF)

S4 Fig The serial bias caused by ambiguous pitches (2–4). (**A**) Pitch serial bias (past-trial pitches = f2, f3, and f4; aggregate results across participants). “High” category choice percent as a function of the current pitch, for different pitch in previous trial (Light-to-dark color lines denote low-to-high pitches). Each circle represents aggregated data for each condition. Solid lines represent the logistic regression fits. (**B**) Category choice serial bias (past-trial pitches = f2, f3, and f4; aggregate results across participants). “High” category choice percent as a function of the current pitch, for different category choice in the previous trial (light green: low category choice; dark green: high category choice). (**C**) Model comparison results (past-trial pitches = f2, f3, and f4). Left: ΔAIC of Model 2 (blue; current trial + previous pitch), Model 3 (green; current trial + previous category choice), and Model 4 (grey; current trial + previous pitch + previous category choice), compared to Model 1 (current trial only). Right: Regression coefficients for previous pitch (blue) and previous category choice (green) extracted from the winning model (Model 4, * in model comparison). Error bars represent 95% confidence interval. (**D**) Grand average decoding performance for past-trial features (past-trial pitches = f2, f3, and f4) as a function of time following the sustained white noise, for pitch (blue) and category choice (green). The pure tone (blue rectangle) was embedded in a 2.25-s sustained white noise (grey horizontal line). Vertical dashed lines from left to right denote the tone onset and response–cue frame, respectively. Horizontal colored lines denote significant temporal clusters (cluster-based permutation test, *p* < 0.001, one-sided, corrected) for each feature. Shadows represent SEM. (**E**) Grand average neural representational shift (Shift_dist) by past-trial features (past-trial pitches = f2, f3, and f4) as a function of time relative to the white noise onset, for pitch (blue) and category choice (green). Positive and negative values correspond to attractive and repulsive direction, respectively. Horizontal colored lines denote significant temporal clusters (cluster-based permutation test, one-sided, corrected, *p* < 0.05) for each feature. Shadows represent SEM. Data supporting this figure found here: https://osf.io/4cwv7/?view_only=3a4885189ebf46aaacf05ef109821d03.
(TIF)

S5 Fig Alpha and beta power decoding performance of previous features and the behavioral relevance. (**A**) Alpha power decoding performance of previous features and the behavioral relevance. Left: Grand average alpha power decoding performance for previous features as a function of time following white noise onset (vertical dotted line), for pitch (blue), category choice (green), and motor response (orange). The vertical dashed line denotes the tone onset. Horizontal colored lines denote significant temporal clusters (cluster-based permutation test, two-sided, corrected, *p* < 0.05) for each feature. Shadows represent SEM. Right: Participants were divided into two groups based on the serial bias behavior in category choice or motor response, respectively. Grand average alpha power decoding performance of High-bias (colored lines) and Low-bias (black line) groups for category choice (upper), and motor response (lower). Horizontal lines denote significant temporal clusters (cluster-based permutation test, one-sided, corrected, *p* < 0.05). (**B**) Beta power decoding performance of previous motor response and the behavioral relevance. Left: Grand average beta power decoding performance for previous motor response as a function of time following white noise onset. Right: Grand average beta power decoding performance of High-bias (orange line) and Low-bias (black line) groups. Horizontal lines denote significant temporal clusters (cluster-based permutation test, one-sided, corrected, *p* < 0.05). Data supporting this figure found here: https://osf.io/4cwv7/?view_only=3a4885189ebf46aaacf05ef109821d03.
(TIF)

## Abbreviations

AIC: Akaike information criterion
EEG: electroencephalography
GLMM: generalized linear mixed-effects model
ICA: independent component analysis
ITI: intertrial interval
RSA: Representational Similarity Analysis
TMS: transcranial magnetic stimulation
WM: working memory
