# Supplementary figures and images for: Feature-specific reactivations of past information shift current neural encoding thereby mediating serial bias behaviors
Source: PLoS Biol. 2023 Mar 24;21(3):e3002056. doi: 10.1371/journal.pbio.3002056 (PMC10075471; doi:10.1371/journal.pbio.3002056)

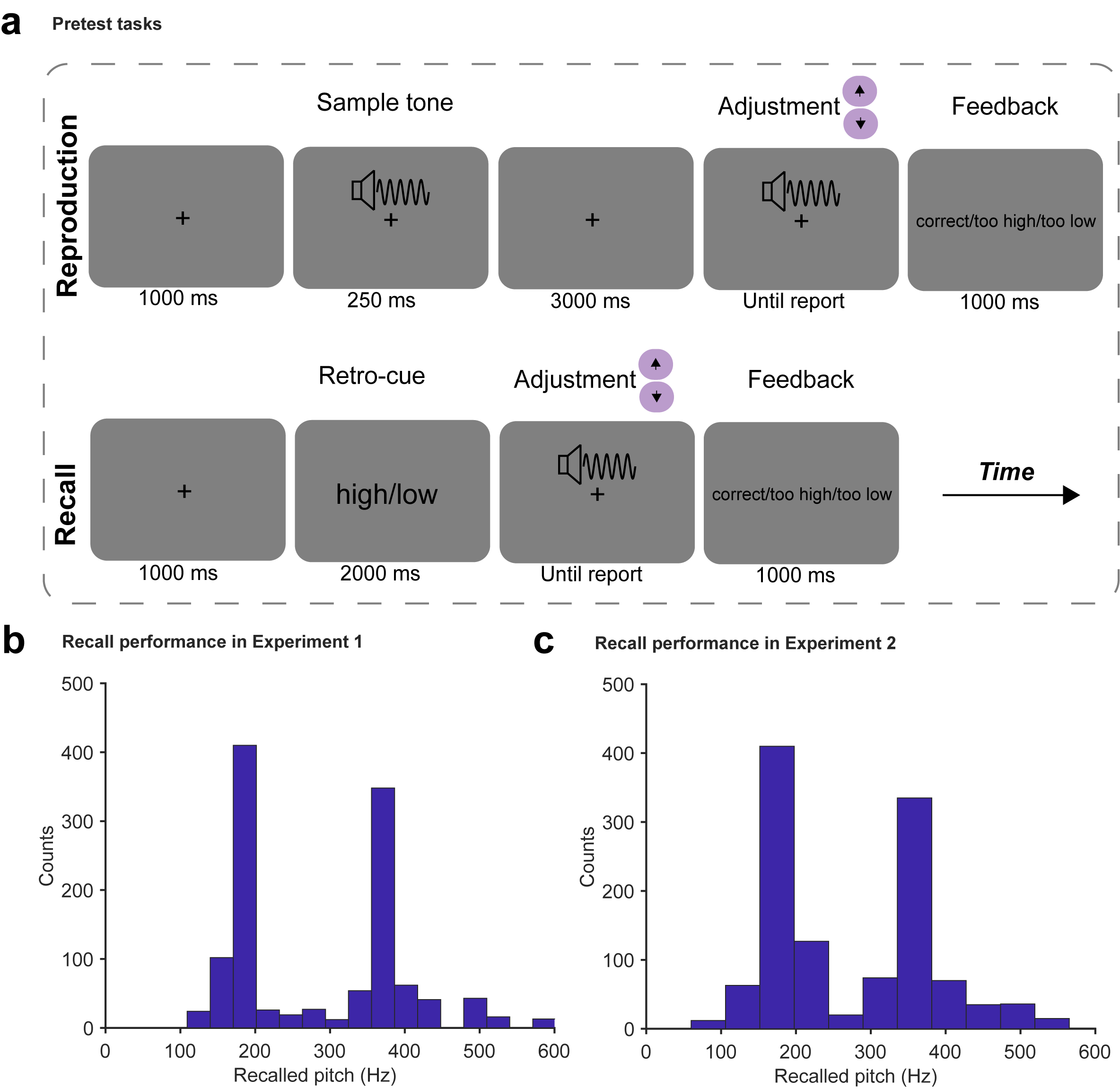

Supplement: S1 Fig — (A) Tone memory task paradigm. Upper: A reproduction task where participants were required to reproduce the given tone (180 or 360 Hz) by using the up and down arrow keys, after which feedbacks were provided. Lower: A recall task where participants had to recall the respective tone according to the retro-cue “high” (360 Hz) or “low” (180 Hz) and reproduce it. The pretest contains a block-design session (two reproduction blocks and two recall blocks each of which testing only one tone for 10 trials) and a randomization design session (one reproduction block and one recall block each of which testing two tones in a trial-by-trial random manner, 20 trials for each tone). (B, C) Recall performance in the randomization design session of Experiments 1 (B) and 2 (C). Both plots show two clear peaks around 180 Hz and 360 Hz (aggregate results across participants). Data supporting this figure found here: https://osf.io/4cwv7/?view_only=3a4885189ebf46aaacf05ef109821d03. (TIF) [file pbio.3002056.s001.tif]

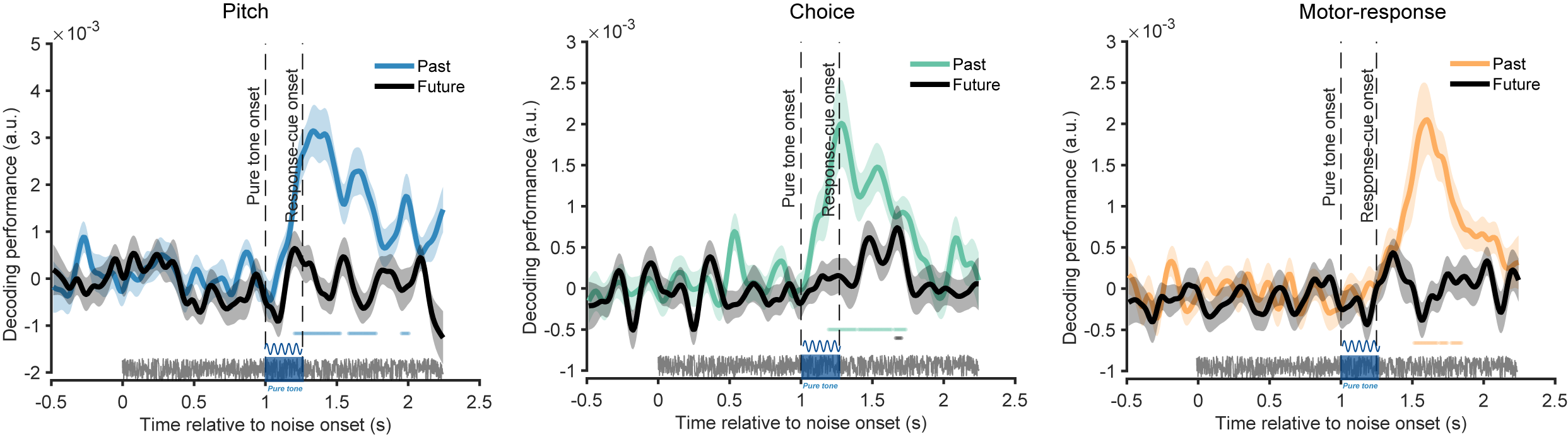

Supplement: S2 Fig — Grand average decoding performance for previous features (in color) and future features (in black) as a function of time following white noise onset, for pitch (left), category choice (middle), and motor response (right). The vertical dashed lines denote the tone onset and the response–cue onset. Horizontal colored lines denote significant temporal clusters (cluster-based permutation test, two-sided, corrected, p < 0.05) for each past feature, and the horizontal black line in the middle panel indicates a marginal significant temporal cluster (cluster-based permutation test, two-sided, corrected, p = 0.056). Shadows represent SEM. Data supporting this figure found here: https://osf.io/4cwv7/?view_only=3a4885189ebf46aaacf05ef109821d03. (TIF) [file pbio.3002056.s002.tif]

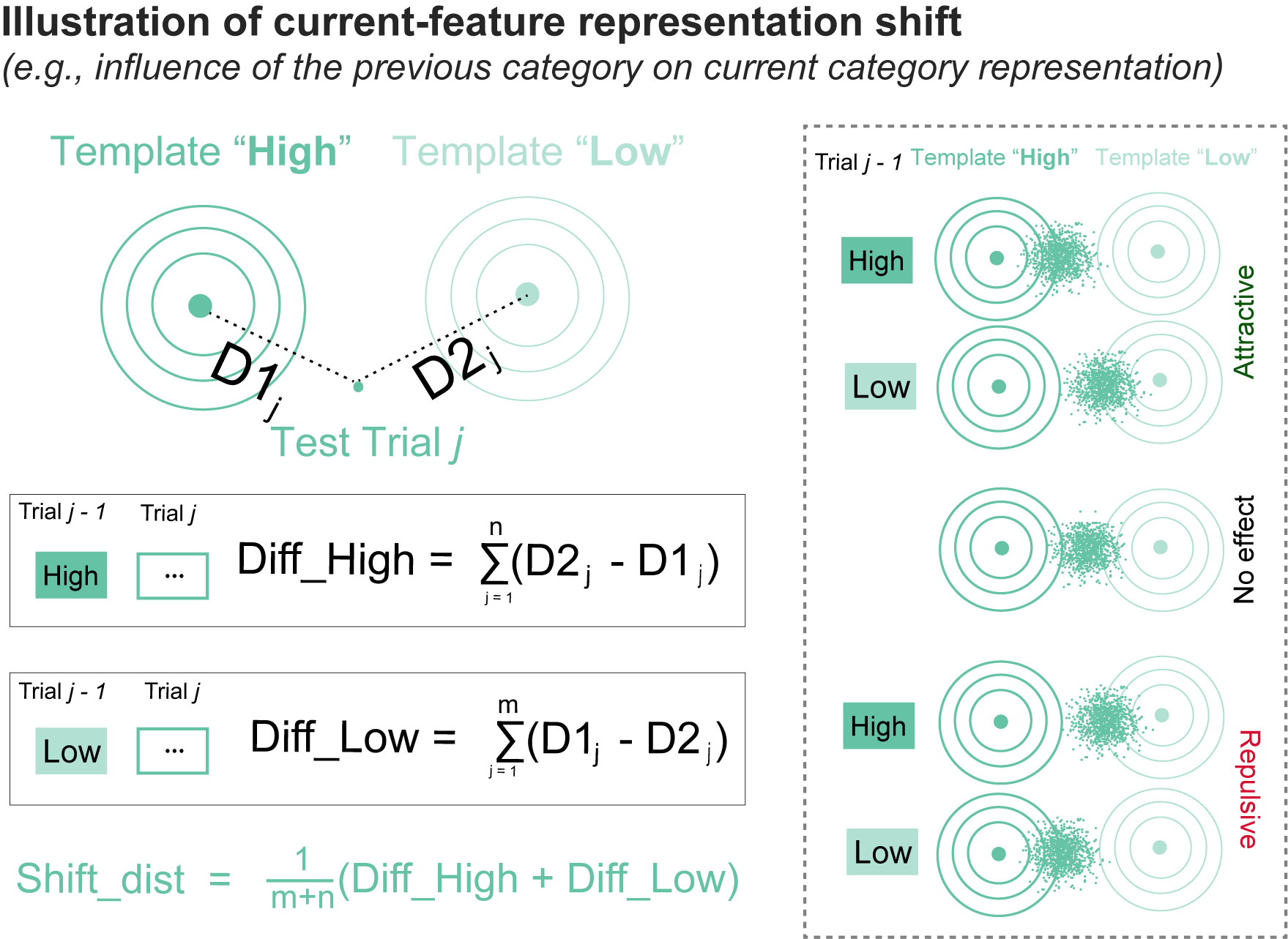

Supplement: S3 Fig — Left: Two neural templates were built for “high” (dark green circle) and “low” (light green circle) category choice base on all trials. For each trial, its neural distance to the two templates were computed, resulting in D1 and D2, respectively. Positive serial bias would predict neural attraction to previous category choice, i.e., positive Diff_High (D2-D1) and positive Diff_Low (D1-D2) values when preceded by “high” and “low” category choice, respectively. The two values were averaged as Shift_dist to characterize the neural shift for category choice. Right: Neural representation of current-trial category is attracted toward (upper, positive Shift_dist values), repulsed from (lower, negative Shift_dist values), or not affected (middle, around zero Shift_dist values) by prior category information. (TIF) [file pbio.3002056.s003.tif]

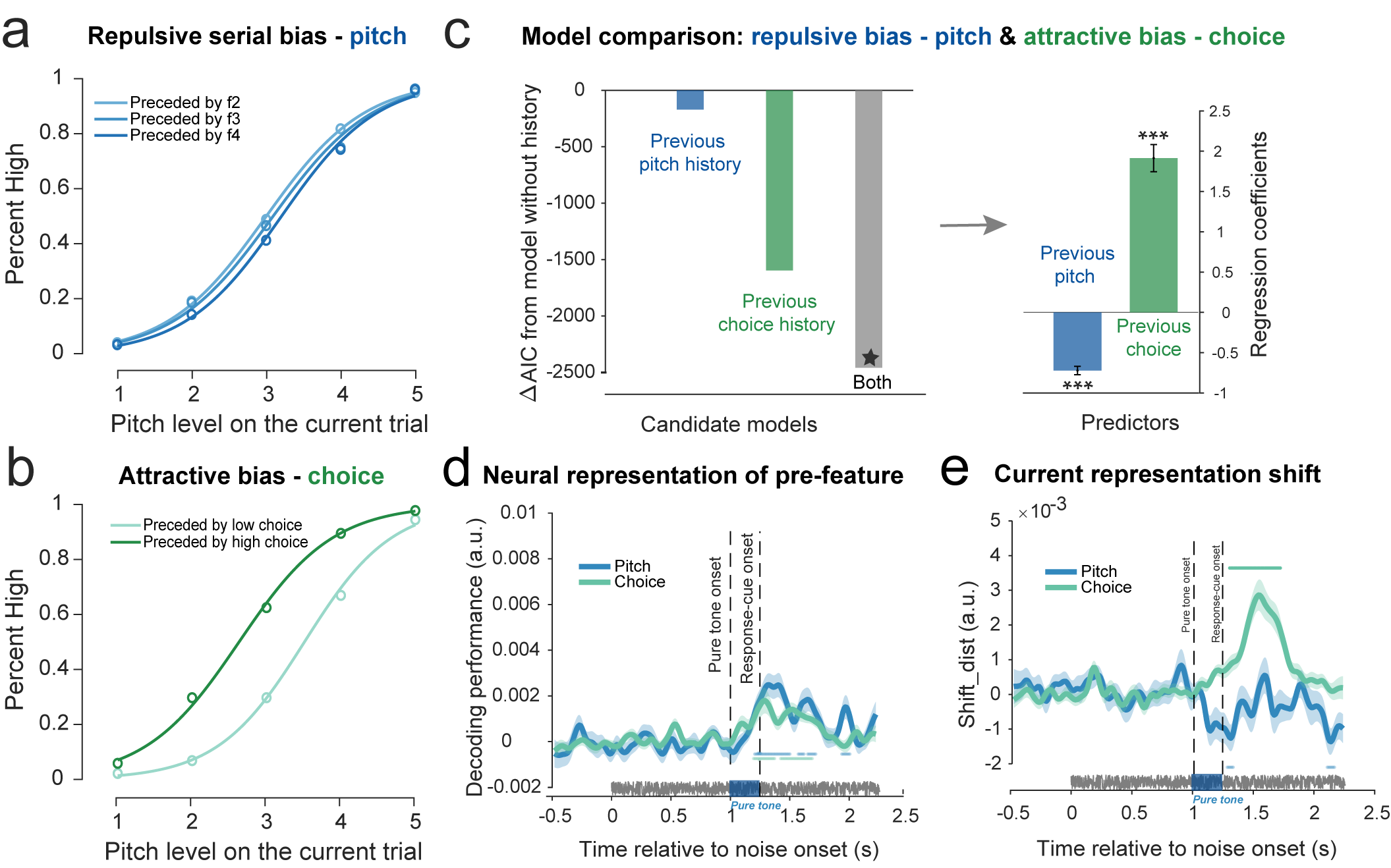

Supplement: S4 Fig — (A) Pitch serial bias (past-trial pitches = f2, f3, and f4; aggregate results across participants). “High” category choice percent as a function of the current pitch, for different pitch in previous trial (Light-to-dark color lines denote low-to-high pitches). Each circle represents aggregated data for each condition. Solid lines represent the logistic regression fits. (B) Category choice serial bias (past-trial pitches = f2, f3, and f4; aggregate results across participants). “High” category choice percent as a function of the current pitch, for different category choice in the previous trial (light green: low category choice; dark green: high category choice). (C) Model comparison results (past-trial pitches = f2, f3, and f4). Left: ΔAIC of Model 2 (blue; current trial + previous pitch), Model 3 (green; current trial + previous category choice), and Model 4 (grey; current trial + previous pitch + previous category choice), compared to Model 1 (current trial only). Right: Regression coefficients for previous pitch (blue) and previous category choice (green) extracted from the winning model (Model 4, * in model comparison). Error bars represent 95% confidence interval. (D) Grand average decoding performance for past-trial features (past-trial pitches = f2, f3, and f4) as a function of time following the sustained white noise, for pitch (blue) and category choice (green). The pure tone (blue rectangle) was embedded in a 2.25-s sustained white noise (grey horizontal line). Vertical dashed lines from left to right denote the tone onset and response–cue frame, respectively. Horizontal colored lines denote significant temporal clusters (cluster-based permutation test, p < 0.001, one-sided, corrected) for each feature. Shadows represent SEM. (E) Grand average neural representational shift (Shift_dist) by past-trial features (past-trial pitches = f2, f3, and f4) as a function of time relative to the white noise onset, for pitch (blue) and category choice (green). Positive [file pbio.3002056.s004.tif]

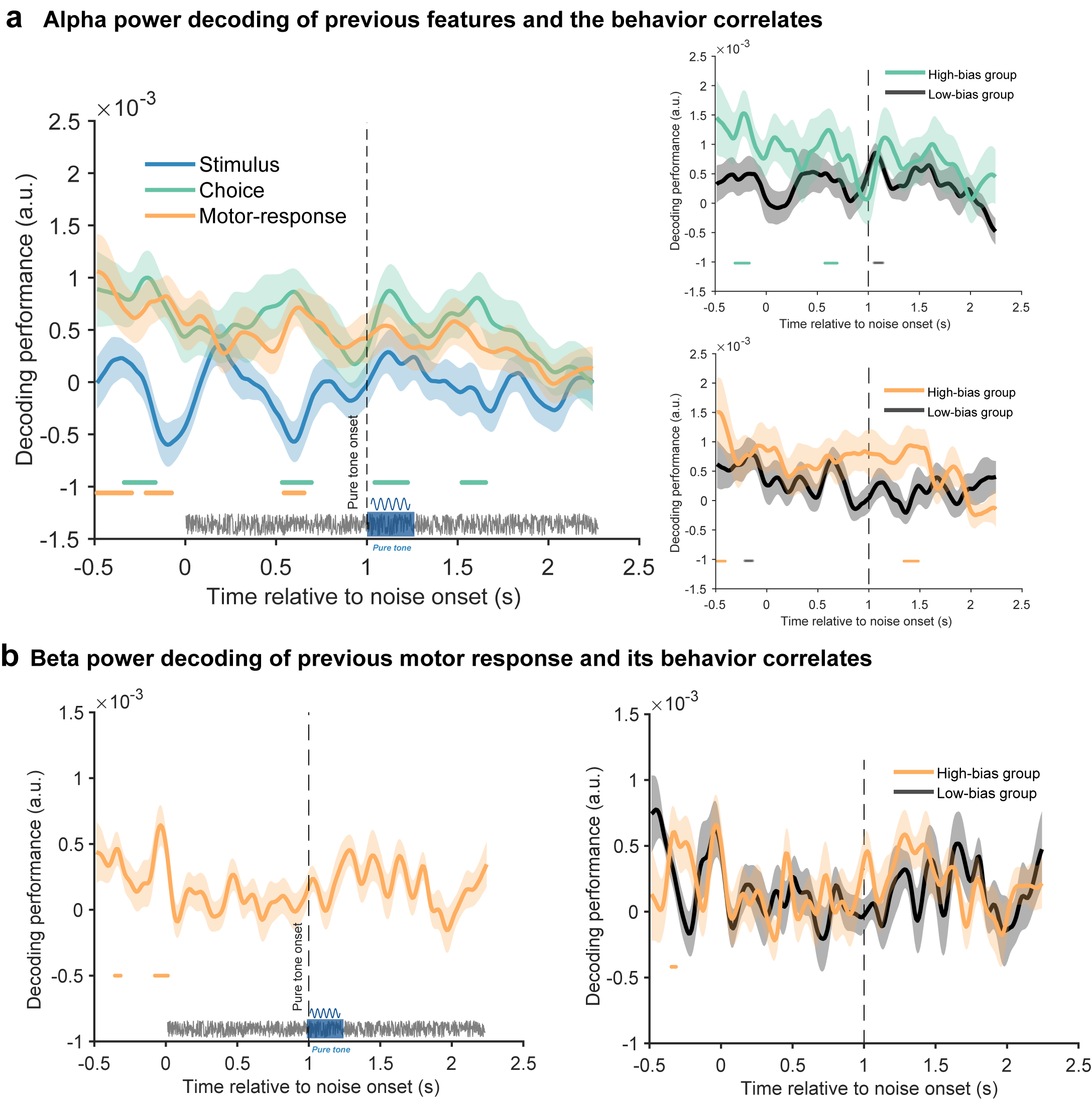

Supplement: S5 Fig — (A) Alpha power decoding performance of previous features and the behavioral relevance. Left: Grand average alpha power decoding performance for previous features as a function of time following white noise onset (vertical dotted line), for pitch (blue), category choice (green), and motor response (orange). The vertical dashed line denotes the tone onset. Horizontal colored lines denote significant temporal clusters (cluster-based permutation test, two-sided, corrected, p < 0.05) for each feature. Shadows represent SEM. Right: Participants were divided into two groups based on the serial bias behavior in category choice or motor response, respectively. Grand average alpha power decoding performance of High-bias (colored lines) and Low-bias (black line) groups for category choice (upper), and motor response (lower). Horizontal lines denote significant temporal clusters (cluster-based permutation test, one-sided, corrected, p < 0.05). (B) Beta power decoding performance of previous motor response and the behavioral relevance. Left: Grand average beta power decoding performance for previous motor response as a function of time following white noise onset. Right: Grand average beta power decoding performance of High-bias (orange line) and Low-bias (black line) groups. Horizontal lines denote significant temporal clusters (cluster-based permutation test, one-sided, corrected, p < 0.05). Data supporting this figure found here: https://osf.io/4cwv7/?view_only=3a4885189ebf46aaacf05ef109821d03. (TIF) [file pbio.3002056.s005.tif]
